# Acute Kidney Injury After CT in Emergency Patients with Chronic Kidney Disease: A Propensity Score-matched Analysis

**DOI:** 10.5811/westjem.2021.1.50246

**Published:** 2021-04-02

**Authors:** Mamata Kene, Vignesh A. Arasu, Ajit K. Mahapatra, Jie Huang, Mary E. Reed

**Affiliations:** * The Permanente Medical Group, Oakland, California; † Kaiser Foundation Hospital, Department of Emergency Medicine, Fremont, California; ‡ Kaiser Foundation Hospital, Department of Radiology, Vallejo, California; § Kaiser Permanente Division of Research, Oakland, California; ¶ Kaiser Foundation Hospital, Department of Nephrology, Santa Clara, California

## Abstract

**Introduction:**

Acute kidney injury (AKI) after intravenous contrast administration for computed tomography (CT) occurs infrequently, but certain patients may be susceptible. This study evaluated AKI incidence among emergency department (ED) patients with pre-existing chronic kidney disease (CKD) undergoing CT exams.

**Methods:**

This retrospective cohort study in an integrated healthcare system included ED patients previously diagnosed with CKD stages 3–5 (estimated glomerular filtration rate <60 milliliters per minute per 1.73 meters squared over at least three months), undergoing CT exams with or without intravenous contrast, from January 1, 2013–December 31, 2017. We excluded patients with CT prior to (30 days) or following (14 days) index CT and missing serum creatinine (sCr) measurements. We applied propensity score matching, and then multivariable regression adjustment for post-CT ED disposition and ED diagnosis, to calculate adjusted risk of AKI. Secondary patient-centered outcomes included 30-day mortality, end-stage renal disease (ESRD) diagnosis, and dialysis initiation.

**Results:**

Among 103,573 eligible ED patients undergoing CT, propensity score matching yielded 5,589 pairs. Adjusted risk ratio (ARR) for AKI was higher overall for contrast-enhanced CT (1.60; 95% confidence interval [CI], 1.43–1.79). However, secondary outcomes were infrequent: 19/5,589 non-contrast vs 40/5,589 contrast patients with new dialysis initiation at 30 days (adjusted risk 0.3% vs 0.7%; adjusted risk reduction 0.4%; 95% CI, 0.1%–0.7%).

**Conclusion:**

In ED patients with chronic kidney disease undergoing CT, intravenous contrast was associated with higher overall adjusted risk of AKI, but patient-centered secondary outcomes were rare. The clinical significance of transient kidney injury after CT is unclear, although patients with advanced chronic kidney disease appear to have elevated risk.

## INTRODUCTION

Increasing use of computed tomography (CT) in United States emergency departments (ED) brings controversy over contrast-associated acute kidney injury (CA-AKI) in focus for ED patients, where a subset may be vulnerable even if overall risk is low.^1^–^8^ The recent American College of Radiology and National Kidney Foundation joint consensus statement suggests for patients with “severe kidney disease,” risks of contrast media are uncertain, and existing evidence may be underpowered to estimate risk of injury.^9^

Early studies overestimated CA-AKI incidence, while recent work casts doubt on the phenomenon of CA-AKI altogether.^8^,^10^–^12^ Meta-analyses concluding that intravenous (IV) contrast is not associated with AKI were not focused on ED patients or chronic kidney disease (CKD), and where CKD patients were included, definitions were inconsistent.^13^–^15^ One meta-analysis included six studies that defined CKD differently, using baseline serum creatinine [sCr] within 24 hours of CT (potentially reflecting AKI rather than CKD), or prior kidney disease diagnoses from the medical record.^7^,^14^,^16^ Chronic kidney disease requires presence of objective laboratory markers of decreased renal function persistent over three months or more; so equating abnormal baseline sCr with CKD may cause misidentification.^17^

Why might CA-AKI go undetected? Studies may be underpowered to detect CA-AKI; substantial confounding may persist despite mitigation attempts, with non-contrast control groups at higher risk for AKI; retrospective cohorts with complete sCr measurements may be sicker overall; undiagnosed AKI could coincide with CT; and propensity score matching may not completely adjust for differences between contrast and non-contrast groups.^18^ Still, propensity score matching may be the most feasible means to evaluate CA-AKI, absent prospective trials.^19^ Finally, the significance of short-term AKI is unclear with respect to patient-centered clinical outcomes such as progression to dialysis and of kidney disease severity.

Among patients with moderate-to-severe CKD (stage 3–5), limited data are reported, yet these patients may be most vulnerable to CA-AKI even if overall risk is low.^7^,^8^,^16^ Two of the largest retrospective CA-AKI studies included small numbers of CKD patients spread over long study periods.^7^,^8^ A study focused on patients with pre-existent CKD might be able to clarify the association of IV contrast with AKI in this potentially at-risk population. We compared the incidence of AKI in a large cohort of ED patients with pre-existing CKD 3–5, undergoing non-contrast or contrast-enhanced CT, by applying propensity scores to match the groups for likelihood of receiving contrast based on presence of previously described AKI risk factors.

## METHODS

### Study Design and Setting

We conducted a retrospective cohort study within Kaiser Permanente Northern California, a large, not-for-profit integrated healthcare system caring for four million patients, with over 1.2 million ED visits annually in 21 community EDs. Patients are similar to the regional population and are socioeconomically, racially and ethnically diverse.^20^ The health system employs a single electronic health record (EHR). The Kaiser Permanente Northern California Institutional Review Board granted a waiver of informed consent for this data-only Health Insurance Portability and Accountability Act-compliant study.

### Data Sources

All data was electronically extracted from the EHR (Epic Systems Corporation, Verona, WI) and its databases by an experienced programmer (JH). Structured electronic extraction used current procedural terminology, internal and *International Classification of Diseases*, *Ninth Revision* and *Tenth Revision* (ICD-9 and ICD-10) codes.

**Population Health Research Capsule**

What do we already know about this issue?

*Contrast computed tomography (CT) may not pose large risk for acute kidney injury (AKI), yet patients with underlying renal dysfunction may be vulnerable to AKI after contrast exposure.*

What was the research question?

*Among patients with chronic kidney disease (CKD), is contrast CT associated with higher incidence of AKI?*

What was the major finding of the study?

*Despite elevated AKI risk in CKD patients undergoing contrast CT, short-term dialysis starts and mortality were uncommon.*

How does this improve population health?

*While contrast CT was associated with elevated AKI risk for CKD patients, the significance of transient AKI after CT is unclear, warranting further study.*

### Participant Selection

All ED visits by adult patients (>17 years) with EHR diagnosis of CKD stage 3–5 who underwent a CT head, neck, chest, abdomen, or pelvis in the ED from January 1, 2013–December 31, 2017 were included.^16^,^17^,^21^ Chronic kidney disease stages 3, 4 and 5 are defined as estimated glomerular filtration rate (eGFR) between 30–59 milliliters per minute per 1.73 meters squared (mL/min/1.73m^2^), 15–29 mL/min/1.73m^2^ and <15 mL/min/1.73m^2^, respectively, persisting over three months or longer. Patients with end-stage renal disease (ESRD) or dialysis were excluded since sCr fluctuations can be inaccurate; we did include patients with CKD stage 5 who were not on dialysis. Patients missing initial and follow-up (24–72 hour) sCr were excluded.^7^,^8^,^10^ We also excluded exams 30 days prior to and 14 days after the index ED visit to avoid confounding due to repeat contrast administration or residual contrast effects. Only the first study-eligible ED CT was included to avoid sampling bias due to clustering by patient; thus, no patients crossed over.

### Exposure Variable

We electronically extracted IV contrast administration based on CT order and procedure code. Omnipaque 300 and 350 and Isovue 370 (non-ionic low-osmolar contrast media) were in use, and institutional protocols recommended administration volumes of 100–150 mL with 20–25% dose reduction for eGFR< 45 mL/min/1.73m^2^, at the discretion of local radiologists and emergency physicians. The range of contrast dose was 75–150 mL, including angiogram (aorta and pulmonary angiogram) studies; given that all contrast was intravenously administered, these studies were considered equivalent for the purpose of renal exposure to contrast. Although the contrast phase for image capture may be different in various studies, this difference should not affect the circulation or renal filtration of contrast. As head, neck, chest, abdomen, and pelvis CTs obtained in the ED may be performed with or without contrast, these studies were included.

We were not able to stratify by body group, similarly to prior studies of AKI after contrast. However, in an attempt to minimize selection bias without excessively restricting the cohort, we excluded extremity CTs as they are rarely performed with IV contrast and would be unrepresented in the contrast group. Contrast-enhanced CT (CECT) was any study or series of studies with IV contrast. Oral contrast administration was not assessed. Consecutive non-contrast CT exams constituted a non-contrast exposure. Our institution does not administer multiple consecutive IV contrast boluses. Two physicians (MVK, emergency medicine; VAA, radiology) reviewed a random sample of imaging orders and reports to validate electronic contrast ascertainment.

### Other Variables and Definitions

We applied propensity score matching to balance for characteristics that may be associated with contrast administration and AKI in the non-contrast and contrast groups. Numerous previously described AKI risk factors were included in the propensity model, including the following: age; gender; ethnicity/race; comorbidities; CKD stage; acute illness severity indicators; and use of potentially nephrotoxic medications (Table 1).^7^, ^8^, ^10^,^18^, ^22^,^23^ Acute illness severity markers were defined as ED systolic blood pressure < 90 millimeters of mercury (mm Hg) and Emergency Severity Index (ESI) level 1 or 2 (ESI is a measure of ED patient acuity^24^). The Modification of Diet in Renal Disease equation was used to calculated eGFR.^25^ Prophylactic medications and IV hydration have been described as of limited use and unclear efficacy, thus were not evaluated.^10^,^14^,^26^

We electronically extracted variables included in the propensity model based on diagnoses, except for hemoglobin and sCr, which were laboratory values. If a patient did not have EHR documentation of a specific diagnosis or medication, they were considered not to have evidence of the condition or medication. Patients with missing sCr values were excluded as this variable was essential to calculating the primary outcome of AKI. For hemoglobin, however, presence of a measured value of hemoglobin < 11milligrams per deciliter (mg/dL) was considered evidence of anemia. No measurements or measurements of hemoglobin ≥ 11 mg/dL were considered absence of evidence of anemia.

In the model to calculate propensity score, we included only factors that could have impacted contrast administration by the emergency physician and would be available *at the time* of the CT and contrast order (treatment assignment). Two important variables associated with AKI that might not be present at the time of the contrast decision are intensive care unit (ICU) admission and admitting diagnosis (sepsis, acute myocardial infarction and multiorgan failure, ICD-9 or 10 codes). We extracted these variables but analyzed them after propensity score modeling.

### Outcome Measures

We calculated the primary outcome, AKI, from sCr values as defined by Acute Kidney Injury Network criteria (absolute sCr increase 0.3 mg/dL or a 1.5-fold increase over baseline sCr), over 24–72 hours after CT, consistent with prior studies.^7^,^10^,^27^–^29^ Given that AKI is not necessarily associated with permanent changes in renal function, we also evaluated secondary patient-centered outcomes. These secondary outcomes (30-day dialysis initiation, new ESRD diagnosis, and mortality) were extracted from the EHR, Social Security Administration, and California state death files. The follow-up window was short to limit confounding by clinical events downstream of the contrast/CT exposure. This study was not designed specifically to detect these secondary outcomes.

### Statistical Analysis

Given practical and ethical concerns of prospective studies precluding randomization for contrast indication, we applied a propensity score-matching approach.^7^,^10^,^16^,^30^,^31^ We calculated the propensity score by using a logistic regression model including characteristics (Table 1) that may influence the decision to administer IV contrast (treatment assignment) and are associated with AKI.^7^,^8^,^10^,^18^,^22^,^23^ Propensity-matched cohorts of a CECT group and a non-contrast CT group were derived by applying 1:1 ratio greedy matching on propensity score, with a caliper of 0.05 standard deviation of the propensity score logit with no replacement. We examined the standardized differences and variance ratios to determine that the matched sample was balanced in patient characteristics. We also graphically examined the distribution of the estimated propensity score for the two groups for the overlap assumption.

In the propensity score-matched sample, we used logistic regression to examine the association between contrast CT and the primary outcome and secondary outcomes adjusted for ED disposition and diagnosis. We calculated the adjusted risk for both groups by applying the coefficients from the multivariable logistic regression model to the study cohort as if every patient were in the CECT group, and every patient were in the non-contrast group, respectively, and reported the adjusted risk differences and risk ratios. Subgroups of CKD severity were evaluated similarly with separate multivariate logistic regression models for CKD stage 3 and CKD stages 4–5.

Since eGFR fluctuates more than CKD stage, we performed a series of sensitivity analyses to evaluate for differences in AKI between contrast and non-contrast groups based on eGFR, a more acute measurement of kidney function. We compared AKI incidence stratified by baseline eGFR (<30, 30–44 and >44 mL/minute (min)/1.73 meters squared [m^2^]) in the original propensity-matched cohort. We also repeated the analyses in three separately propensity score-matched cohorts by baseline eGFR strata.

All analyses were conducted with SAS version 9.4 (SAS Institute, Inc., Cary, NC) and Stata version 14.2 (StataCorp, College Station, TX). Statistical significance level was set at *P*-value <.05.

## RESULTS

### Study Subject Characteristics

During the study period, 103,573 adult ED patients with CKD stages 3–5 underwent eligible CT studies. After excluding 10,938 patients with preceding (30 days prior) and 4,918 with subsequent (14 days after) CT, removing patients with missing baseline (11,771) and follow-up (49,031) sCr values, and restricting the cohort to the first eligible visit (excluding 5,178 encounters) in the study period, 21,737 encounters remained, with 5,980 CECT and 15,757 non-contrast CT (Figure). Propensity score matching yielded 5,589 pairs of patients (391 patients from the CECT group were excluded because there was no match in the non-contrast CT group). The characteristics of the two groups were balanced with the absolute value of standardized difference <0.10 and variance ratios between 0.5 and 2.0. There was no evidence of violation of the overlap assumption when checking the distributions of propensity scores of the two groups (Appendix A).

Characteristics of the original and propensity-matched populations are presented in Table 1, including age, gender, race/ethnicity, pre-CT sCr (laboratory measurement within 24 hours prior to CT), ICD-9 or 10 diagnoses (proteinuria, hypoalbuminemia, single kidney, renal transplant, peripheral vascular disease, coronary artery disease, history of myocardial infarction, diabetes, congestive heart failure, hypertension), anemia (laboratory measurement hemoglobin <11 mg/dL) and outpatient prescription (past 90 days) or ED use of nephrotoxic medications (diuretic, angiotensin-converting enzyme inhibitor, antimicrobial agents, non-steroidal anti-inflammatories, others – Appendix B). Older age, non-white race, male gender, and comorbidities except peripheral vascular disease and hypoalbuminemia were significantly associated with non-contrast CT. All variables in Table 1 were included in the propensity model.

We identified 5,589 pairs of patients with CECT and non-contrast CTs using propensity score matching, median age 80 years for non-contrast CT (interquartile range 72–86 years) and 79 years for CECT (interquartile range 72–85 years) exams. Comorbidity and demographic characteristics were comparable between groups in the propensity score matched cohort (Table 1). Diabetes mellitus, hypertension, and anemia were prevalent. After propensity score matching, CKD stage 4 or 5 was present in 3% of the cohort.

Patients in the non-contrast group were more likely to be admitted to the ICU (9% vs 7%, 510 of 5,589 non-contrast patients and 383 of 5,589 CECT patients, respectively, *P*<0.001) and had a higher frequency of acute organ failure (65 of 5,589 vs 39 of 5,589, *P* = 0.01), whereas the CECT group had a higher frequency of acute heart failure diagnosis (6% or 326 of 5,589 CECT patients vs 4% or 217 of 5,589 non-contrast patients, *P*<0.001) (Table 2). The frequency of acute myocardial infarction (2%, 83 of 5,589 non-contrast patients and 2% or 90 of 5,589 CECT patients, *P* = 0.59) and sepsis (6% or 332 of 5,589 non-contrast patients and 6% of 315 of 5,589 CECT patients, *P* = 0.49) were not different between groups.

### Primary Outcome AKI Incidence

After propensity score matching, the adjusted risk of AKI was 8.3% in the non-contrast group compared to 13.2% for CECT for 5,589 pairs (adjusted risk ratio [ARR] for AKI 1.60, 95% confidence interval [CI], 1.43–1.79) (Table 3). The AKI absolute risk difference was 5% higher for CECT (95% CI, 3.8%–6.1%). The higher risk of AKI in the CECT remained significant in the stratum of patients with CKD stage 3 (7.9% non-contrast vs 12.8% CECT for 5403 pairs, ARR 1.61, 95% CI,1.43–1.80) but not for the smaller stratum of CKD 4–5 patients (18.9% non-contrast vs 26.8% CECT for 186 pairs, ARR 1.41, 95% CI, 0.96–2.08). Unadjusted incidence of AKI is available in Appendix C, Table C1.

### Secondary Patient-centered Outcomes

Adjusted risks for secondary patient-centered outcomes at 30 days (new diagnosis of ESRD, initiation of dialysis, and mortality) are reported in Table 4. New initiation of renal dialysis and new diagnosis of ESRD were rare (Appendix C, Table C2). Both non-contrast and CECT groups had notable 30-day mortality (8.5% and 7.1%, respectively).

### Sensitivity Analysis

The results from sensitivity analyses separately analyzing AKI incidence stratified by baseline pre-CT eGFR in the propensity-matched cohort as well as in a separately propensity-matched cohort based on eGFR strata (45–59, 30–44 and <30 ml/min/1.73m^2^) were consistent with the results based on CKD stage (3 vs 4–5) (Appendix C, Tables C3 and C4).

## DISCUSSION

In a study of contrast CT and acute kidney injury among ED patients with chronic kidney disease in an integrated healthcare system, we found that IV contrast-enhanced CT was associated with increased overall risk of AKI compared to non-contrast CT (adjusted risk difference 5%, 95% CI, 3.8%–6.1%; ARR 1.60, 95% CI, 1.43–1.79). Secondary patient-centered outcomes (mortality, new dialysis initiation) were rare, limiting conclusions about the difference between groups; however, the overall low observed frequency at 30 days suggests need for further study of any relationship between AKI in the setting of IV-contrast administration and clinically meaningful outcomes.

Most prior contrast-associated AKI studies were not focused on CKD patients or emergency patients, but recent literature calls for further knowledge in patients with “severe kidney disease” in whom prior studies have reached differing conclusions.^3^,^7^,^9^,^16^ Meta-analyses conclude no association between contrast and AKI, but one study points out a major risk factor for AKI after contrast is pre-existent chronic kidney dysfunction, which is not uniformly treated across studies.^14^,^21^,^27^ Accurate risk characterization is important in these patients, to consider whether to employ dose reduction, to avoid contrast, or to consider alternatives to CT. We focused on CKD patients evaluated in the ED, where urgent diagnostic evaluation requires contrast administration in many cases; we applied propensity score matching to mitigate selection bias in contrast administration and adjusted for post-CT acute illness factors.

The small number of propensity matched pairs with severe CKD in our study and others points to CECT avoidance despite literature suggesting negligible overall CA-AKI incidence. Few studies have focused specifically on CKD patients, and varying results are reported in subsets of larger studies, with inconsistent definitions of renal dysfunction that do not distinguish between abnormal “baseline” pre-CT eGFR etiologies – whether due to incipient AKI, chronically abnormal eGFR without ongoing AKI, or concurrent AKI and CKD at the time of the study.^7^,^8^,^10^,^32^ Including patients with incipient AKI or undiagnosed renal dysfunction may obscure AKI ascertainment.

Comparing results across studies with different definitions of abnormal renal function is also difficult. Hinson et al reported 1557 patients (12%) with CKD diagnosis in a larger study yet almost double – 3021 (23%) – the number of CKD patients had eGFR <60 ml/min/1.73m^2^ at the time of CT, suggesting a notable degree of unexplained renal dysfunction in the cohort, while Davenport et al included 3685 patients (20%) with eGFR<60 ml/min/1.73m^2^ and excluded patients with undefined “unstable renal function.”^7^,^10^ McDonald et al studied 1220 propensity matched pairs with eGFR<60 ml/min/1.73m^2^, requiring two available sCr values 24 hours prior to CT, potentially selecting for sicker patients.^16^ Of these studies, only Davenport et al identified increased AKI odds for CECT among patients with eGFR<30 ml/min/1.73m^2^ (2.96; 95% CI, 1.22–7.17).^7^ In our study, CKD stage aligned closely but not perfectly with baseline eGFR, and sensitivity analyses of separately derived eGFR cohorts were consistent with CKD stage-based findings. The small subgroup of severe CKD or very low eGFR suggests that patients with very abnormal renal function may be unlikely to receive IV contrast, and statistical power was limited in this subgroup in our study.

Patient-centered outcomes of new dialysis, ESRD, and mortality are difficult to evaluate because confounding increases with time after contrast exposure yet are clinically important. Measured changes in renal function may lag behind physiological injury,^33^ yet the definition of AKI relies on serial sCr measurements; this difficulty applies to all investigations of AKI and highlights the importance of evaluating clinical and patient-centered outcomes alongside laboratory values. We observed infrequent new dialysis initiation and new ESRD diagnosis, possibly related to a small event rate limiting statistical power, coding lags, and imbalance in unmeasured confounders. In a meta-analysis of AKI and secondary outcomes, mortality odds were similar (0.998, 95% CI, 0.730–1.362) among all patients, yet CKD patients may have elevated mortality risk regardless of CT.^14^

Mortality in our cohort was notable for both non-contrast and CECT, likely due to selecting for availability of serial sCr measurements. A recent review underscores this difficulty in retrospectively understanding transient sCr changes, secondary patient-centered outcomes, and the relationship between the two, suggesting that measuring renal injury related to contrast is limited both by the questionable significance of transient post-CT sCr changes and by possible confounding in reported longer term outcomes.^34^ The observations in the current study of the low secondary-outcome frequencies despite the noted incidence of AKI ranging from 8.3% (non-contrast) to 13.2% (CECT) suggests that AKI may not translate into clinically important renal injury after IV contrast. Study of alternate outcomes such as 30-day renal function recovery or strategies to predict AKI risk and need for post-CT renal function monitoring may be more clinically relevant. A prospective study or a much larger sample would be necessary to accurately evaluate these patient-centered outcomes.

## LIMITATIONS

Our study had several limitations. Inclusion and eligibility criteria limited our study cohort. Although we could not adjust for CT indication, propensity score matching may be the most feasible retrospective approach to balance for treatment assignment (contrast); IV contrast is filtered by the kidneys similarly regardless of indication for IV contrast. However, a retrospective approach cannot discriminate between the potential effect of contrast and the disease process identified by the CT exam. Intra-arterial contrast was not studied in this investigation. We took care to select CT studies that are performed with and without IV contrast in the absence of a prospective study that would allow some form of randomization to contrast, and excluded extremity CTs, which are typically non-contrast studies. Completeness of sCr values was limited, similar to previous studies, and might be differentially measured after CT in sicker patients.^7^,^8^,^10^,^28^ We addressed the potential for undiagnosed renal dysfunction by measuring pre-CT sCr and matching for renal function at the time of CT. The CKD 4–5 subgroup illustrates difficulties in retrospectively balancing contrast: CKD 4–5 prevalence was 3% (186 CECT and 186 non-contrast) in the propensity-matched cohort, and the study lacked power to separately assess this group.

The small number of patients in the most severe kidney disease (CKD 4–5) subgroup resulted in inadequate discriminatory power to ascertain AKI risk, yet these findings suggest that clinicians avoid IV- contrast exposure in patients with severe kidney disease even if prior literature suggests negligible risk of AKI, and that post-contrast outcomes in patients with baseline renal dysfunction warrant further study. We may not have captured all relevant covariables in this retrospective electronic extraction, but we included many described AKI risk factors; therefore, we do not expect that our study was more subject to these biases than previous similar investigations.^7^,^8^,^10^,^11^,^18^,^21^

## CONCLUSION

In summary, we observed increased overall risk of acute kidney injury after contrast CT in this cohort of patients with known chronic kidney disease. The substantial attrition in our and other studies, combined with our findings of higher acute kidney injury risk among contrast-exposed patients with chronic kidney disease, suggest that prospective studies in this specific subpopulation are needed. While randomization is unlikely, prospectively recruiting patients undergoing CT would facilitate serial serum creatinine measurements and evaluation of meaningful outcomes like 30-day renal function recovery.

## Supplementary Information



## Figures and Tables

**Figure:**
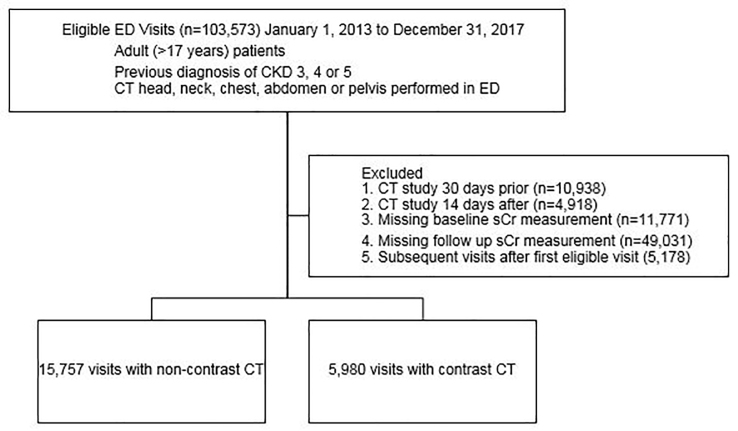
Cohort Derivation: Adult (age>17 years) emergency department (ED) patients with chronic kidney disease (CKD) stages 3–5 undergoing computed tomography (CT) (head, neck, chest, abdomen, pelvis) January 1, 2013 to December 31, 2017.

**Table 1:** Characteristics of the original and 1:1 propensity-matched population; all variables included in propensity model.

	Original cohort				Propensity matched cohort			
	
	Non-contrast n (%)	CECT n (%)	Standardized difference	Variance ratio	Non-contrast n (%)	CECT n (%)	Standardized difference	Variance ratio
N	15,757 (100)	5,980 (100)			5,589 (100)	5,589 (100)		
eGFR (ml/min/1.73m^2^)								
<30	5,292 (34)	100 (2)	−0.92	0.07	98 (2)	100 (2)	0.00	1.02
30–<45	5,155 (33)	1,211 (21)	0.29	0.73	1,212 (22)	1,205 (22)	0.00	1.00
45–59	5,310 (34)	4,669 (78)	−1.00	0.77	4,279 (77)	4,284 (77)	0.00	1.00
Age (years)								
<65	1,800 (11)	685 (11)	0.00	1.00	575 (10)	614 (11)	−0.02	1.06
65–<75	3,180 (20)	1,489 (25)	−0.11	1.16	1,224 (22)	1,224 (22)	0.00	1.00
75–<85	5,464 (35)	2,228 (37)	−0.14	0.97	2,181 (39)	2,181 (39)	0.00	1.00
85+	5,313 (34)	1,578 (26)	−0.16	0.87	1,609 (29)	1,570 (28)	−0.02	0.99
Gender								
Male	7,375 (47)	2,239 (37)	0.19	0.94	2,223 (40)	2,143 (38)	0.03	0.99
Race or ethnicity								
White	9,733 (62)	4,089 (63)	0.14	0.92	3,780 (68)	3,782 (68)	0.00	1.00
Black	1,917 (12)	552 (9)	0.10	0.78	545 (10)	538 (10)	0.00	0.99
Hispanic	1,910 (12)	660 (11)	0.03	0.92	620 (11)	624 (11)	0.00	1.01
Asian	1,988 (13)	593 (10)	0.09	0.81	572 (10)	568 (10)	0.00	0.99
Other	209 (1)	86 (1)	−0.01	1.08	72 (1)	77 (1)	−0.01	1.07
Comorbidity								
CKD 4–5	2,825 (18)	213 (4)	0.05	0.90	186 (3)	186 (3)	0.01	0.97
Coronary artery disease	2,181 (14)	734 (12)	0.21	0.79	726 (13)	704 (13)	0.00	1.00
Congestive heart failure	4,622 (29)	1,226 (21)	0.48	0.23	1,194 (21)	1,193 (21)	0.00	1.00
History of myocardial infarction	2,570 (16)	800 (13)	0.08	0.85	781 (14)	774 (14)	0.00	0.99
Hypoalbuminemia	86 (1)	23 (0)	0.02	0.71	20 (0)	23 (0)	−0.01	1.15
Proteinuria	1,081 (7)	259 (4)	0.11	0.65	242 (4)	246 (4)	0.00	1.02
Renal transplant	155 (1)	14 (0)	0.10	0.24	13 (0)	14 (0)	0.00	1.08
Single kidney	240 (2)	64 (1)	0.04	0.71	55 (1)	58 (1)	0.00	1.05
Peripheral vascular disease	3,083 (20)	1,143 (19)	0.01	0.98	1,042 (19)	1,074 (19)	−0.01	1.02
Anemia (lab)	9,150 (58)	2,812 (47)	0.22	1.02	2,669 (48)	2,653 (48)	0.01	1.00
Hypertension	14,299 (91)	5,311 (89)	0.06	1.18	4,996 (89)	4,986 (89)	0.01	1.01
Diabetes mellitus	7,614 (48)	2,538 (42)	0.12	0.98	2,410 (43)	2,382 (43)	0.01	1.00
Nephrotoxic medications								
ACE-I	6,019(38)	2,665 (45)	−0.13	1.05	2,451 (44)	2,471 (44)	−0.01	1.00
Diuretic	6,041(38)	2,101 (35)	−0.05	1.04	1,924 (34)	1,979 (35)	−0.02	1.02
Antimicrobial	4,726(30)	1,936(32)	0.07	0.96	1,760 (32)	1,810 (32)	−0.02	1.01
NSAID	706 (5)	400(7)	−0.10	1.46	333 (6)	351 (6)	−0.01	1.05
Other Nephrotoxic	5,221(33)	1,917(32)	0.02	0.98	1,718 (31)	1,779 (32)	−0.02	1.02
Severity in ED								
ED SBP<90	841 (5)	249 (4)	0.06	0.79	195 (4)	212 (4)	−0.01	1.08
ESI level 1–2	5,514 (35)	1,847 (31)	0.09	0.94	1,821 (33)	1,753 (31)	0.03	0.98

*CECT*, contrast-enhanced computed tomography; *eGFR*, estimated glomerular filtration rate; *CKD*, chronic kidney disease; *mL*, milliliters; *min*, minute; *m^2^*, meters squared; *ACE-I*, ace inhibitor; *NSAID*, non-steroidal anti-inflammatory drug.

*ED*, emergency department; *SBP*, systolic blood pressure; *ESI*, Emergency Severity Index.

**Table 2:** Post-computed tomography and post-contrast characteristics of acuity.

	Propensity matched cohort		

	No contrast n (%)	CECT n(%)	P-value
ED disposition			
Total	5,589 (100)	5,589(100)	
ICU admission	510 (9)	383 (7)	<0.0001
Hospital admission	4,179 (75)	4,309 (77)	
Discharged	900 (16)	897 (16)	
ED diagnosis			
Acute heart failure	217 (4)	326 (6)	<0.0001
AMI	83 (2)	90 (2)	0.59
Sepsis	332 (6)	315 (6)	0.49
Multiorgan failure	65 (1)	39 (1)	0.01

* Based on ICD-9 and 10 diagnosis codes for index visit

*CECT*, contrast-enhanced computed tomography; *ED*, emergency department; *ICU*, intensive care unit; *AMI*, acute myocardial infarction.

**Table 3:** Adjusted* risk of acute kidney injury in propensity matched cohort, overall and stratified by chronic kidney disease stage.

	Total (n)	Adjusted risk**	Adjusted risk difference (95% CI)** for CECT - non-contrast CT	Adjusted risk ratio (95% CI) for CECT/non-contrast CT
Overall				
Non-contrast	5,589	8.3%		
CECT	5,589	13.2%	5.0% (3.8%–6.1%)	1.60 (1.43–1.79)
CKD stage 3				
Non-contrast	5,403	7.9%		
CECT	5,403	12.8%	4.8% (3.7%–6%)	1.61 (1.43–1.80)
CKD stage 4–5				
Non-contrast	186	18.9%		
CECT	186	26.8%	7.8% (0.7%–16.4%)	1.41 (0.96–2.08)

* Adjusted for post-computed tomography and post-contrast acuity characteristics (emergency department disposition to intensive care unit and ED diagnosis of acute myocardial infarction, sepsis or multi-organ failure)

** Rounded to single decimal point

*CI*, confidence interval; *CECT*, contrast-enhanced computed tomography; *AKI*, acute kidney injury; *CKD*, chronic kidney disease.

**Table 4:** Adjusted* risk of secondary patient-centered outcomes.

	Adjusted risk	Adjusted risk difference for CECT/Non-contrast (95% CI)	Adjusted risk ratio for CECT/Non-contrast (95% CI)
30-day new initiation of dialysis			
Non-contrast	0.3%		
CECT	0.7%	0.4% (0.1%–0.7%)	2.14 (1.24–3.70)
30-day ESRD diagnosis			
Non-contrast	0.6%		
CECT	0.9%	0.2% (0%–0.5%)	1.39 (0.89–2.17)
30-day mortality			
Non-contrast	8.5%		
CECT	7.1%	−1.4% (−2.0– −0.4%)	0.84 (0.74–0.95)

* Adjusted for post-computed tomography and post-contrast acuity characteristics (Emergency department (ED) disposition to intensive care unit and ED diagnosis of acute myocardial infarction, sepsis or multi-organ failure).

*CECT*, contrast-enhanced computed tomography; *CI*, confidence interval; *ESRD*, end-stage renal disease.
