# Supplementary material for: Acute Kidney Injury After CT in Emergency Patients with Chronic Kidney Disease: A Propensity Score-matched Analysis
Source: West J Emerg Med. 2021 Apr 2;22(3):614–22. doi: 10.5811/westjem.2021.1.50246 (PMC8203012; doi:10.5811/westjem.2021.1.50246)
Supplement: Supplementary file 1 [file wjem-22-614-s001.docx]

**Appendix A: Overlap distribution of propensity scores in non-contrast and CECT groups**


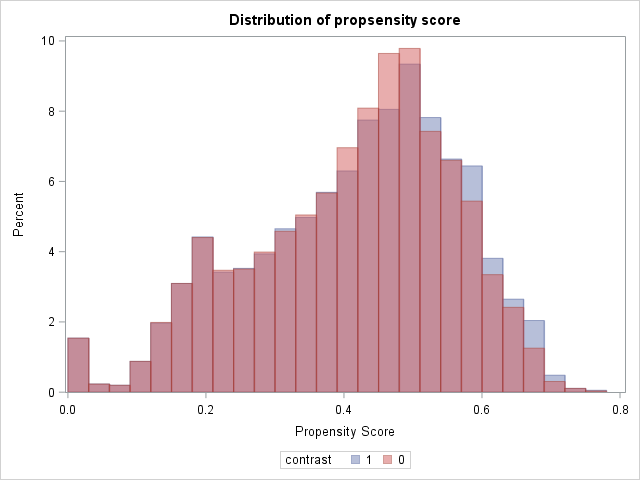


0= non-contrast CT

1= contrast-enhanced CT

**Appendix B: List of potentially nephrotoxic medications**

**ACE inhibitors and Angiotensin Receptor Blockers**

Benazepril

Candesartan

Captopril

Enalapril

Enalaprilat

Irbesartan

Lisinopril

Losartan

Olmesartan

Ramipril

Valsartan

**Antimicrobials**

Acyclovir

Amikacin

Amoxicillin/clavulanic acid

Amphotericin

Ampicillin/sulbactam

Aztreonam

Cefazolin

Cefepime

Cefotaxime

Cefotetan

Cefoxitin

Cefpodoxime

Ceftazidime

Ceftriaxone

Cephalexin

Chloramphenicol

Ciprofloxacin

Cyclosporine

Dicloxacillin

Doxycycline

Fluconazole

Flucytosine

Ganciclovir

Gentamicin

Indinavir

Itraconazole

Levofloxacin

Moxifloxacin

Oxacillin

Penicillin

Piperacillin

Piperacillin/tazobactam

Quinine

Rifampin

Streptomycin

Tenofovir

Tetracycline

Tobramycin

Trimethoprim/sulfamethoxazole

Vancomycin

Voriconazole

**Diuretics (loop and thiazide)**

Furosemide

Torsemide

Chlorothiazide

Chlorthalidone

Hydrochlorothiazide

**Nonsteroidal Anti-inflammatory drugs**

Celecoxib

Diclofenac

Ibuprofen

Indomethacin

Ketorolac

Naproxen

Meloxicam

Piroxicam

Etodolac

Rofecoxib

**Other**

Allopurinol

Amitriptyline

Hydralazine

Lithium

Pantoprazole

Lansoprazole

Omeprazole

Phenytoin

Cimetidine

Ranitidine

Terbutaline

Topiramate

Zonisamide

Cisplatin

Carboplatin

Gemcitabine

Ifosfamide

Methotrexate

Mitomycin

Pentostatin

Tacrolimus

Sirolimus

Everolimus

Mesalamine

**Appendix C: additional tables and sensitivity analyses**

Table C1: Unadjusted incidence of AKI, propensity matched cohort, overall and by CKD stage

|  |  | Propensity matched cohort | | |
| --- | --- | --- | --- | --- |
|  |  | AKI/total (n) | AKI (%) | p-value |
| Overall | non-contrast | 458/5,589 | 8.2% | <0.0001 |
|  | CECT | 746/5,589 | 13.4% |  |
|  |  |  |  |  |
| CKD stage |  |  |  |  |
| CKD 3 | non-contrast | 425/5,403 | 7.9% | <0.0001 |
|  | CECT | 694/5,403 | 12.8% |  |
| CKD4-5 | non-contrast | 33/186 | 17.7% | <0.0001 |
|  | CECT | 52/186 | 28% |  |

AKI = acute kidney injury

CECT = contrast-enhanced CT

CKD = chronic kidney disease

Table C2: Unadjusted incidence of secondary patient-centered outcomes

|  | Propensity-matched cohort | | |
| --- | --- | --- | --- |
|  | Non-contrast | CECT |  |
|  | n (%) | n (%) | p-value |
|  | 5,589 (100) | 5,589 (100) |  |
| 30-day new initiation of dialysis | 19 (0) | 40 (1) | 0.01 |
| 30-day ESRD diagnosis | 34 (1) | 46 (1) | 0.18 |
| 30-day mortality | 481 (9) | 395 (7) | 0.003 |

CECT = contrast-enhanced CT

ESRD = end-stage renal disease

Table C3: Sensitivity analyses: Unadjusted AKI incidence, stratification by eGFR (ml/min/1.73m^2^) and separately derived propensity-matched cohorts by eGFR

|  |  | Original propensity-matched cohort | | | Separately eGFR-based propensity-matched cohort | | |
| --- | --- | --- | --- | --- | --- | --- | --- |
|  |  | AKI/total (n) | AKI (%) | p-value | AKI/total (n) | AKI (%) | p-value |
| eGFR 45-59 | non-contrast | 334/4,279 | 7.8% | <0.0001 | 303/3,970 | 7.6% | <0.0001 |
|  | CECT | 532/4,284 | 12.4% |  | 496/3,970 | 12.5% |  |
| eGFR 30-44 | non-contrast | 110/1,212 | 9.1% | <0.0001 | 104/1,132 | 9.2% | <0.0001 |
|  | CECT | 189/1,205 | 15.7% |  | 178/1,132 | 15.7% |  |
| eGFR <30 | non-contrast | 14/98 | 13.3% | 0.058 | 17/100 | 17% | 0.16 |
|  | CECT | 25/100 | 25% |  | 25/100 | 25% |  |

AKI = Acute kidney injury

eGFR = estimated glomerular filtration rate

CECT = contrast-enhanced CT

Table C4: Adjusted risk of AKI for original propensity-matched subgroups by estimated glomerular filtration rate (ml/min/1.73m^2^) and separately propensity matched cohort by eGFR

|  |  | Total (n) | AKI % | Adjusted Risk Difference  (95% CI) | Adjusted Risk Ratio (95% CI) |
| --- | --- | --- | --- | --- | --- |
| Original propensity-matched cohort | | | | | |
| eGFR 45-59 |  |  |  |  |  |
|  | non-contrast | 4,279 | 7.9% |  |  |
|  | CECT | 4,284 | 12.4% | 4.5% (3.2%-5.8%) | 1.57 (1.38-1.79) |
| eGFR 30-44 |  |  |  |  |  |
|  | non-contrast | 1,212 | 9.2% |  |  |
|  | CECT | 1,205 | 15.5% | 6.3% (3.7%-8.8%) | 1.68 (1.35-2.09) |
| eGFR <30 |  |  |  |  |  |
|  | non-contrast | 98 | 15.0% |  |  |
|  | CECT | 100 | 24.8% | 9.8% (-1.7%-21.3% | 1.65 (0.9-3.03) |
| Separately eGFR-based propensity matched cohort | | | | | |
| eGFR 45-59 |  |  |  |  |  |
|  | non-contrast | 3,970 | 7.7% |  |  |
|  | CECT | 3,970 | 12.4% | 4.8% (3.5%-6.1%) | 1.62 (1.42-1.86) |
| eGFR 30-44 |  |  |  |  |  |
|  | non-contrast | 1,132 | 9.3% |  |  |
|  | CECT | 1,132 | 15.6% | 6.3% (3.7%-9%) | 1.68( (1.34-2.11) |
| eGFR <30 |  |  |  |  |  |
|  | non-contrast | 100 | 17.8% |  |  |
|  | CECT | 100 | 24.5% | 6.6% (-5%-18.3%) | 1.37 (0.78-2.41) |

*Adjusted for post-CT and post-contrast acuity characteristics (ED disposition to ICU and ED diagnosis of acute myocardial infarction, sepsis or multi-organ failure)

AKI = Acute kidney injury

eGFR = estimated glomerular filtration rate

CECT = contrast-enhanced CT
